# Nitrate-Nitrogen Leaching and Modeling in Intensive Agriculture Farmland in China

**DOI:** 10.1155/2013/353086

**Published:** 2013-07-30

**Authors:** Ligang Xu, Hailin Niu, Jin Xu, Xiaolong Wang

**Affiliations:** ^1^State Key Laboratory of Lake Science and Environment, Nanjing Institute of Geography and Limnology, Chinese Academy of Sciences, Nanjing, China; ^2^College of Ecological and Environmental Engineering, Qinghai University, Xining, China; ^3^Department of Environmental Engineering, Nanjing Institute of Technology, Nanjing, China

## Abstract

Protecting water resources from nitrate-nitrogen (NO_3_-N) contamination is an important public health concern and a major national environmental issue in China. Loss of NO_3_-N in soils due to leaching is not only one of the most important problems in agriculture farming, but is also the main factor causing nitrogen pollution in aquatic environments. Three typical intensive agriculture farmlands in Jiangyin City in China are selected as a case study for NO_3_-N leaching and modeling in the soil profile. In this study, the transport and fate of NO_3_-N within the soil profile and nitrate leaching to drains were analyzed by comparing field data with the simulation results of the LEACHM model. Comparisons between measured and simulated data indicated that the NO_3_-N concentrations in the soil and nitrate leaching to drains are controlled by the fertilizer practice, the initial conditions and the rainfall depth and distribution. Moreover, the study reveals that the LEACHM model gives a fair description of the NO_3_-N dynamics in the soil and subsurface drainage at the field scale. It can also be concluded that the model after calibration is a useful tool to optimize as a function of the combination “climate-crop-soil-bottom boundary condition” the nitrogen application strategy resulting for the environment in an acceptable level of nitrate leaching. The findings in this paper help to demonstrate the distribution and migration of nitrogen in intensive agriculture farmlands, as well as to explore the mechanism of groundwater contamination resulting from agricultural activities.

## 1. Introduction

Nitrogen levels in surface water and groundwater of agricultural lands have increased by 50% over the past two decades as a result of increases in the use of fertilizers and manure [1, 2]. As the nation with the largest agricultural production, China consumed 23 million tons of fertilizer N in 2000, accounting for about 28% of total world N consumption [3–5]. In China, the current nitrogen use had led to considerable nitrate-nitrogen (NO_3_-N) losses through leaching. Nitrogen losses through leaching vary across a field due to differences in soil physical properties and N status of soil. The NO_3_-N, a water-soluble nutrient, is transported to shallow groundwater as a leachate that consequently contaminates it. NO_3_-N levels have been of worldwide concern due to the deteriorating quality of groundwater and surface water for the past four decades. As the NO_3_-N exceeded the safe drinking water standards in drinking water, it always experiences some health problems. The major problems linked to NO_3_-N contamination are methemoglobinemia (blue baby syndrome) infants and human birth defects [6]. The concern about the health and environmental effects of NO_3_-N contaminated surface and groundwater has made it imperative to estimate NO_3_-N losses from cropland and to evaluate the impact of crop production practices on NO_3_-N leaching.

Numerical models are useful tools to predict the risk of NO_3_-N′ contamination to surface water and groundwater. These models need to be calibrated and validated for the conditions under which they will be used. A properly validated model provides a fast and cost-effective way of estimating NO_3_-N leaching under different agricultural management practices. Thus, the farmer can more accurately determine the amount of fertilizer to use on a crop to manage yield yet avoid over fertilization, while political decision makers can identify agricultural best practices. The number of nonpoint source agricultural models used to predict NO_3_-N leaching through the root zone and into the underlying unsaturated soil zone has grown rapidly over the last two decades. The creation, calibration, and validation of water quality computer models impact agricultural practices leading to greater awareness and potential control of environmental impacts. These include nitrogen and carbon cycling in soil water and plant (NCSWAP) by Clemente et al. [7], pesticide root zonemodel (PRZM) by Cameira et al. [8], groundwater loading effects of agricultural management system (GLEAMS) by Leonard et al. [9], SLIM by Addiscott [10], nitrate leaching and economic analysis package (NLEAP) by Prasad [11], SOILSOILN by Bergstrom and Jarvis [12], GRAzing SIMulation Model (GRASIM) by Sarmah et al. [13], HYDRUS by Simunek et al. [14], RZWQM (Root ZoneWater Quality Model) by Hu et al. [15], and leaching estimation and chemistry model (LEACHM) by Hutson and Wagenet [16]. Evaluation of these models has also received increasing attention over the last decade [17–21]. If these models are appropriately validated with respect to their simulative capability under various conditions, the models will significantly improve the quantitative understanding of N cycling processes, which can be valuable tools in designing environmentally compatible and economically suitable agricultural systems [5].

Owing to intensive cropping of the orchard and vegetables combined with excessive use of fertilizer in China, the nitrogen pollution has become increasingly serious. However, it is not clear on the quantitive risk and the effects of NO_3_-N leaching in intensive agricultural cropped soil in China. The objectives of this paper deal with (1) to explore the transport and fate characteristics of NO_3_-N within the soil profile and the leaching loss from continuously typical intensive agriculture farmland, (2) to estimate the LEACHM model against the data in terms of its ability to simulate the process of NO_3_-N leaching loss in field conditions, and (3) to determine the overall NO_3_-N leached from the irrigation and natural rainfall and the leaching potential of nitrates under current traditional irrigation methods and suggest the best possible irrigation methods that can reduce nitrogen leaching.

## 2. Materials and Methods

### 2.1. Site Description

**Figure 1:**
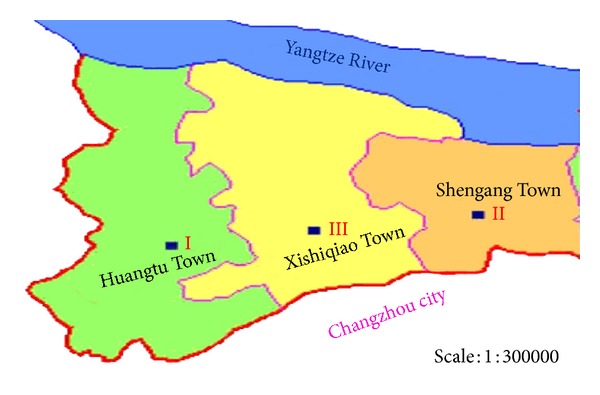
The distribution map of typical agriculture areas in Jiangyin City (I is grape orchard, II is vegetable base, and III is conventional planted farmland).

The experimental site is located at the Qinshuihe catchment of Jiangyin cities through which the Yangtze River flows and has 200 km distance from Shanghai and Nanjing city of China. The average annual precipitation of the site is 1205.5 mm of which about 700 mm occurs during the crop growing period. Jiangyin city's GDP is one of the top three country cities in China. The utilization of agriculture is dominated by intensive cropped type. The risk and environmental effect on intensive agriculture cropped soil, especially on excessive nitrogen fertilization application, are not clear. Three kinds of typical intensive cropped soil were selected in Jiangyin city as the case study for NO_3_-N leaching and modeling study. The first one is a typical grape orchard located in the Huangtu town. The second one is a typical vegetable base located in the Shengang town. The last one is conventional cropped soil in Xishiqiao town. The map for the study area is illustrated in Figure 1. The soil is sampled in three kinds of typical cropped soil, and the basic soil physical properties and background values for soil are given in Table 1.

### 2.2. Microclimate Monitoring and Crop Management

A microclimate monitoring system by the Decagon Device Company was set up in the field to monitor an integrated climate variables (leaf wetness, precipitation, relative humidity, solar radiation, temperature, wind direction, and wind speed). A comprehensive questionnaire was delivered to the local farmers in three kinds of typical intensively cropped farmland on the following: the cropping patterns, crop varieties, seasonal crop inputs, the amount and date of fertilization, irrigation, medicine, and so forth.

### 2.3. Soil Moisture Monitoring and NO_3_-N Sampling

A field experiment is designed to monitor NO_3_-N leaching losses from nitrogen fertilized and manured intensive cropped farmland. The EC-5 soil moisture made by the Decagon Device has been used to monitor the soil moisture in three different soil profiles. In each monitor station, the soil moisture sensors were set up at the depth of 20 cm, 40 cm, 60 cm, 80 cm, and 100 cm, respectively. Hourly data were recorded automatically with 24-hour cycle of each day at the three field stations. These data were used to calibrate soil hydraulic parameters and validate the model. The soil for three kinds of the cropped soil profile (1.0 m thick) was sampled at 0.2, 0.4, 0.6, 0.8, and 1.0 m depths for NO_3_-N analyzing using an automated Cd reduction method (USEPA, 1979).

### 2.4. Groundwater Observation Well Monitoring in Field

**Figure 2:**
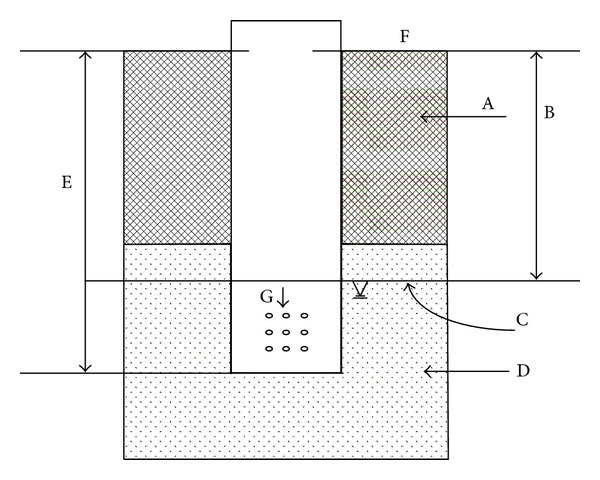
Schematic diagram of groundwater monitoring well (A: soil layer; B: depth of groundwater; C: groundwater level; D: sand layer; E: depth of groundwater monitoring well; F: soil surface; G: infall).

Three groundwater observation monitoring wells were built on three representative cropped farmland. The groundwater observation well is made of PVC with a protective casing on it to prevent the influx of garbage and insects (Figure 2). The groundwater sampling was usually performed once a week, but additional observation was conducted when there was a rainstorm. The water was promptly sent to the laboratory for analysis. Meanwhile, water level and farming conditions (fertilization, irrigation, and crop stage), the growing and physiological character, the yield of plants during their growing time, and crop harvest time were noted when they were happening. NO_3_-N in percolation water was analyzed with a continuous-flow nitrogen analyzer (SKALAR, San Plus System, Netherlands).

### 2.5. Model Selection and Calibration Description

Modeling of water flow movement and NO_3_-N transport vertically in the soil profile was conducted using LEACHM model (Hutson and Wagenet, 1997). This model had been used with varying degrees of success, primarily for determining the magnitude of nitrate leached below the plant root zone. This software package is a one-dimensional model for simulating the transient movement of water and multiple solutes in variably saturated media, with extensive capabilities, such as options to simulate crop root water uptake. It is easy to set flexible boundary conditions, time step, convergence conditions, and the output format for LEACHM, which greatly improves computational efficiency and simulation precision of the model. The LEACHM is selected for this study because it has subroutines to calculate water flow, NO_3_-N leaching, evapotranspiration, rate constant adjustments for temperature and water content, and uptake. The previous studies in China have tested that LEACHM gives a fair description of the NO_3_-N dynamics in the soil as other models (NCSWAP, GRASIM, HYDRUS,…). Currently, LEACHM has been widely used in the research of soil water, salt, and nitrogen transport. It would aid our understanding of the nitrogen migration and cycle. A detailed description of LEACHM can be found in Huston (1996, 2005, and 2009) and others [22, 23].

The soil profile was divided into 10 increments in the vertical direction with a uniform thickness of 0.1 m. The upper boundary condition was set as a fixed flux, while the bottom boundary was defined as a fixed water table depth condition. Each increment required the following data: bulk density, particle size distribution, initial N concentrations, initial soil water content, water retention parameters, saturated hydraulic conductivity, and dispersivity. Values for bulk density and saturated hydraulic conductivity were obtained from laboratory tests. The soil physical properties of each drainage class are presented in Table 1, and other model parameter values used in the simulations are presented in Table 2. The empirical constants of the Campbell retentively function were obtained by log transformation of (1). The only parameter calibrated for nitrogen transport was the dispersion coefficient by comparing the measured data and simulated data. The output time step was set up as 0.01 day.

In order to obtain a quantitative assessment of simulation results, correlation coefficient (*r*) was adopted to evaluate numerical simulation precision:

(1) $$\begin{array}{c} r=\frac{\sum_{i=1}^{N}\left(M_{i}-\bar{M}\right)\left(E_{i}-\bar{E}\right)}{\sqrt{\sum_{i=1}^{N}{\left(M_{i}-\bar{M}\right)}^{2}{\left(E_{i}-\bar{E}\right)}^{2}}}, \end{array}$$

where, *M*_*i*_ and *E*_*i*_ are, respectively, the *i*th measured values and simulated values; *N* is the observation frequency. The value range of correlation coefficient (*r*) is [−1, +1], with a correlation coefficient of +1 indicating that the two variables have a perfect, upward-sloping (+) linear relationship and a correlation coefficient of −1 showing that the two variables have a perfect, downward-sloping (+) linear relationship. A correlation coefficient of 0 stands for nonlinear relationship between the variables. 

## 3. Results and Discussion

### 3.1. NO_**3**_-N Transport and Fate in Soil Profile

**Figure 3:**
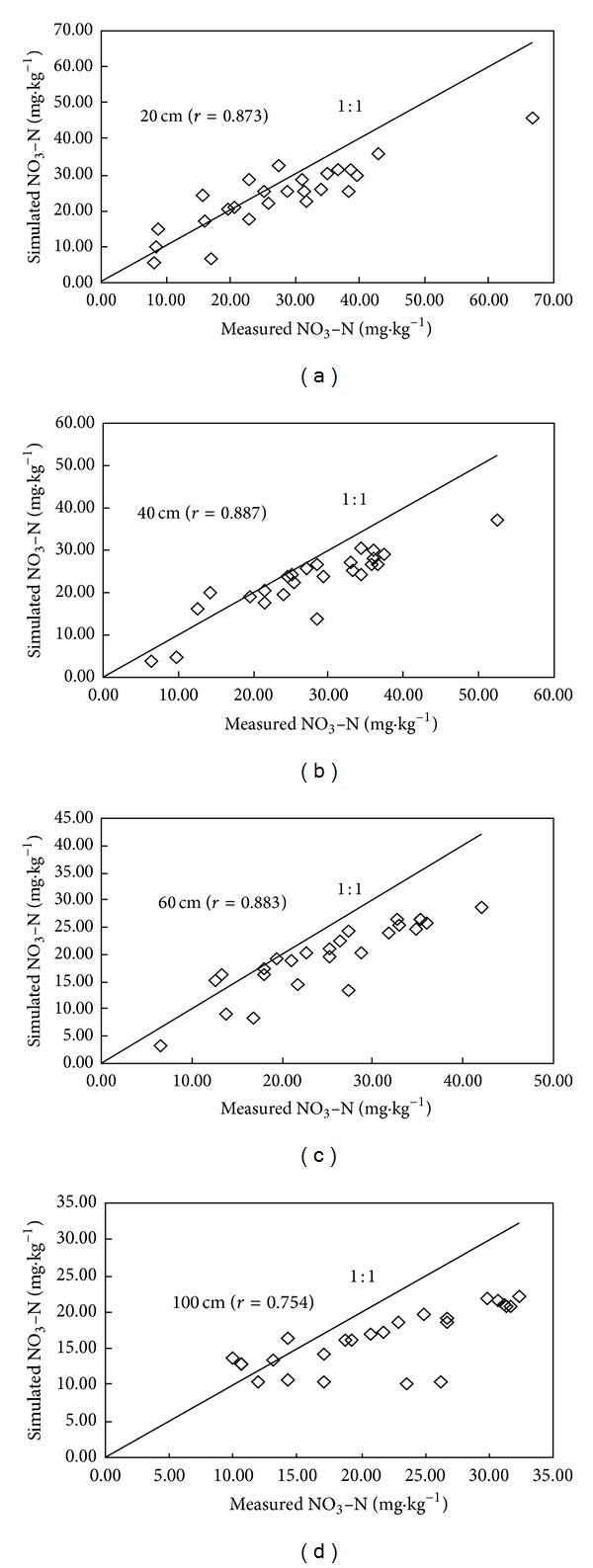
Comparison between simulated values and measured values of NO_3_-N with different soil layer in intensive grape orchard.

**Figure 4:**
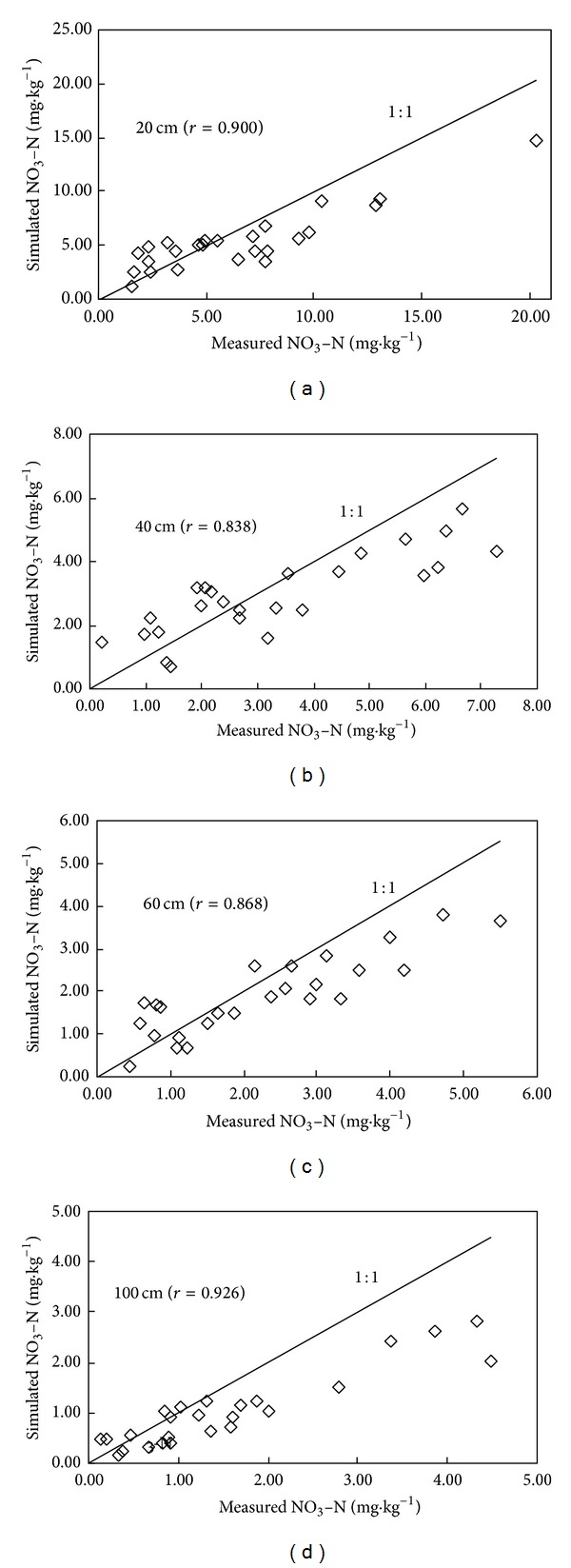
Comparison between simulated values and measured values of NO_3_-N with different soil layer in intensive vegetable base.

**Figure 5:**
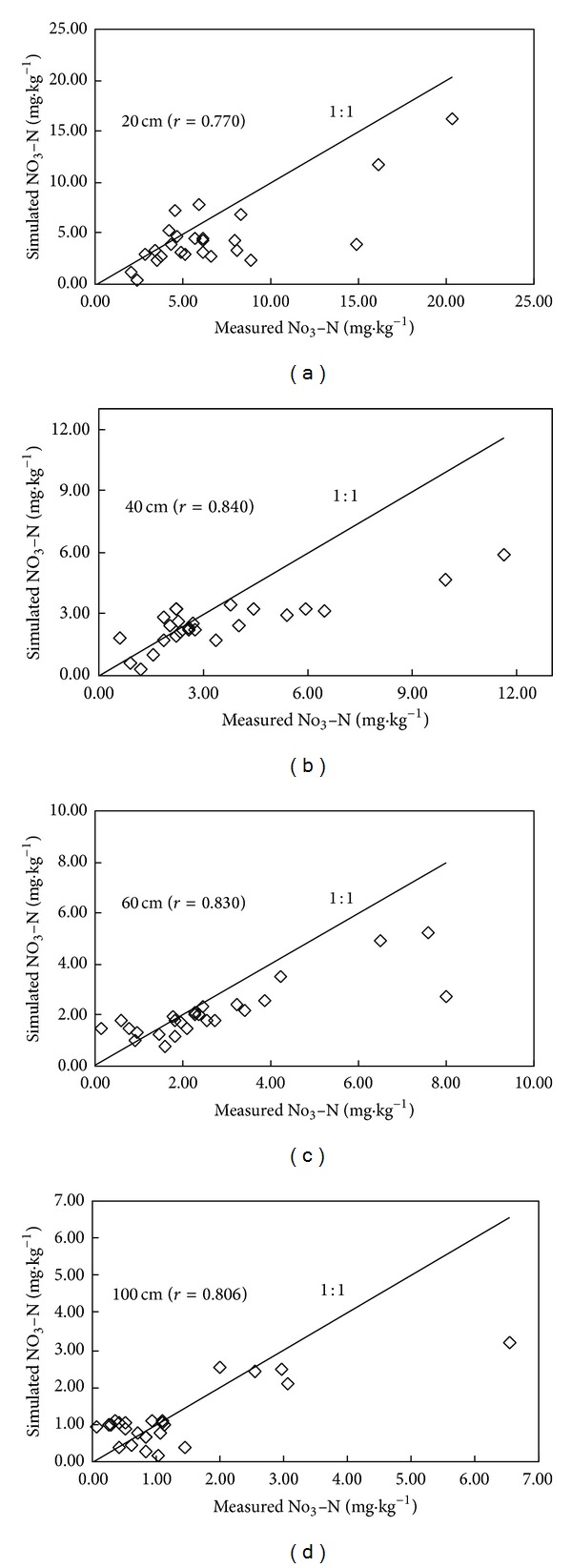
Comparison between simulated values and measured values of NO_3_-N with different soil layer in conventional planted farmland.

Based on the field monitoring, the LEACHM model was tested to simulate the transport and fate of NO_3_-N in soil profile from May 21 to December 8 in 2010. Figures 3, 4, and 5 demonstrate the comparison between simulated values and measured values of NO_3_-N at different depths in three different cropped lands, respectively. The LEACHM model was found to be able to successfully simulate the concentration of NO_3_-N at different depths in soil. The simulations for all three kinds of typical cropped soil provided satisfactory results even through simulated values deviated from measured values in the initial stage. The average correlation analysis for three typical farmlands shows that 20 cm, 40 cm, 60 cm, and 100 cm soil layer between the simulated and measured values are 0.967, 0.942, 0.9243, and 0.893. Correlation coefficient of the soil reaches a significant level. It indicates that the model simulation of NO_3_-N migration performs well. The simulated results reflect the regime of vertical migration of NO_3_-N in soil under normal growing conditions. This discrepancy can be explained by the accumulation of water on the sand layer during the initial stage of the experiment leading to a lag in water drainage and thus leaching loss. 

Overall, the results from this study show that the LEACHM model has the potential to predict the fate of N added to soil in relation to NO_3_-N leaching loss below the 100 cm depth using parameters derived from previous experiments. Further field-testing using data from various soils, crops, management, and weather conditions is needed to evaluate the model's application to different field conditions.

### 3.2. NO_**3**_-N Leaching from Irrigation and Natural Rainfall Condition

**Figure 6:**
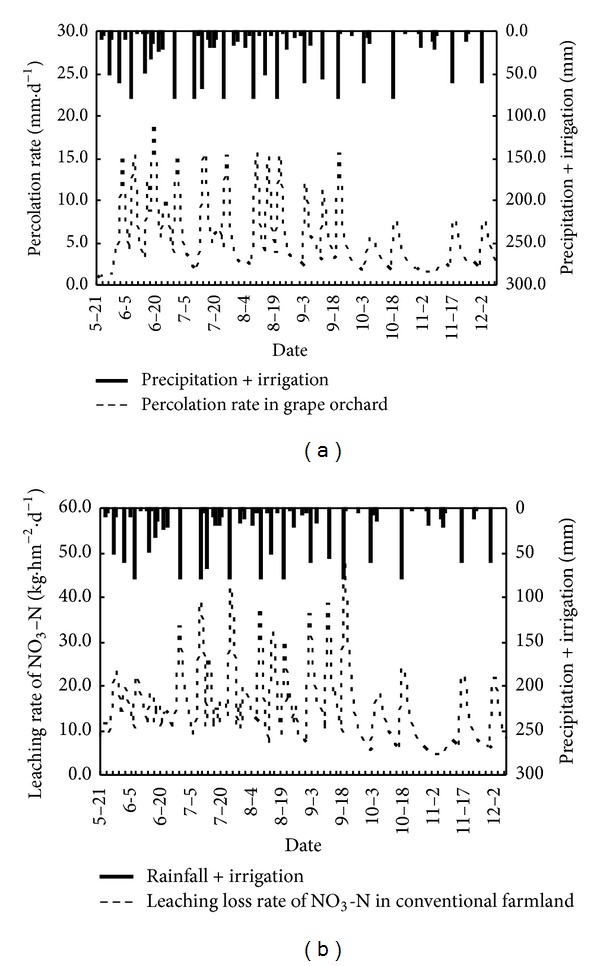
Variation curves of soil water percolation rate and NO_3_-N leaching loss rate with date in intensive grape orchard.

**Figure 7:**
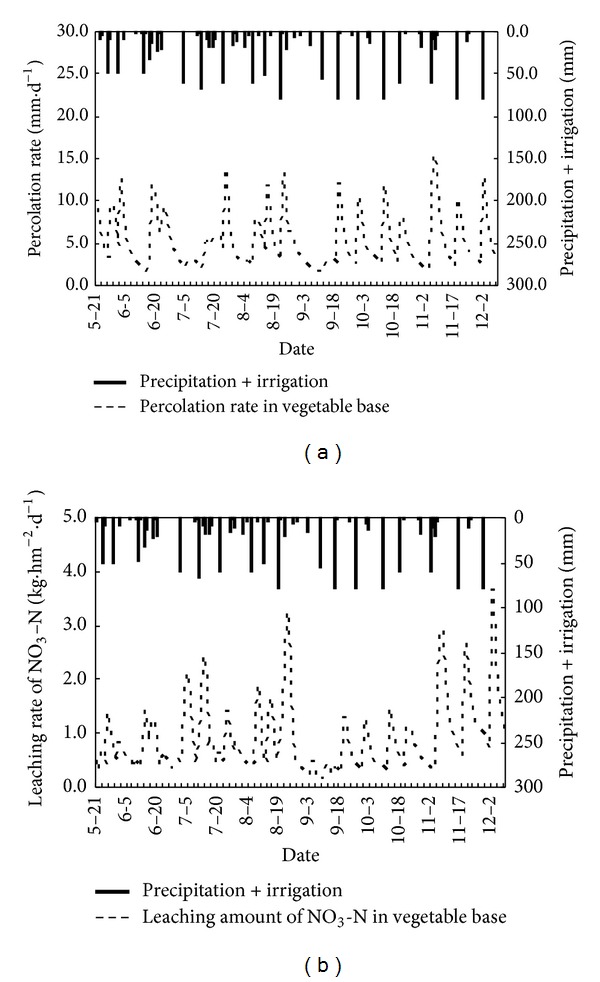
Variation curves of soil water percolation rate and NO_3_-N leaching loss rate with date in intensive vegetable base.

**Figure 8:**
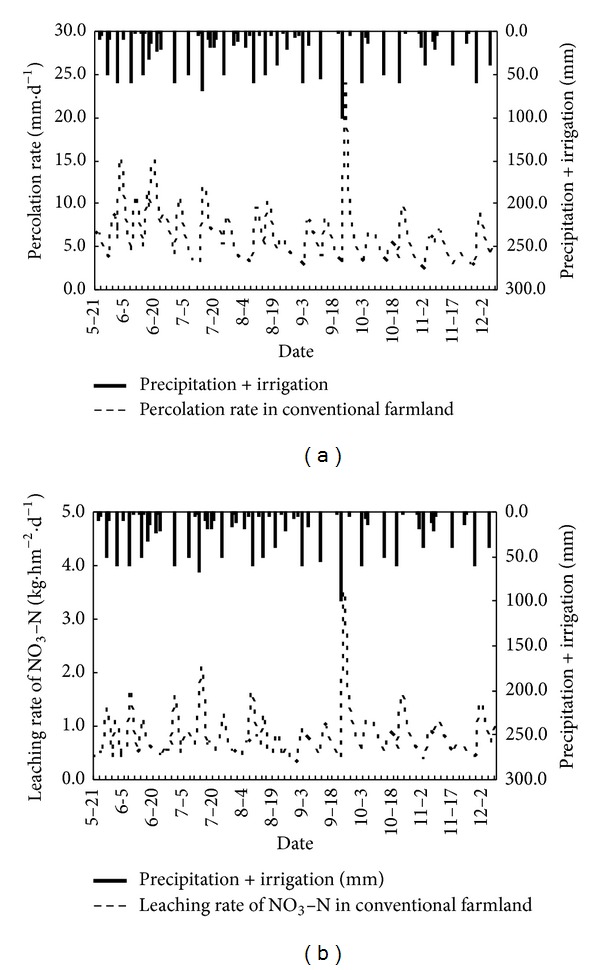
Variation curves of soil water percolation rate and NO_3_-N leaching loss rate with date in conventional farmland.

Soil water percolation rate and NO_3_-N leaching loss rate are shown in Figures 6, 7, and 8, and it can be seen that the simulation results of NO_3_-N for all three different planted farmland exhibit the same trend under different rainfall intensities. The increase of rainfall intensity has a clear positive correlation with leaching loss, such that an increase in rainfall intensity results in a corresponding increasing rate of leaching loss. The measured drainage water is lower than the simulated values in the initial stage of all three sets of experiments. This is due to the effect of the formation of a thin saturated layer at the sand area, which results in a short-term accumulation and lag of water drainage. This explained the lower measured values. After the initial stage, the simulated values and measured values match well and become stable. The simulated results show that the LEACHM model performs well and gives ideal simulations of water drainage in all three sets of experiments under different rainfall intensities.

Table 3 shows the comparison of soil water seepage and soil water storage changes in three typical cropped farmlands. In the conventional cropped farmland, the amount of soil water leakage within 0–100 cm is high as 1177.6 mm. It is significantly higher than the intensive grapes orchard (971.8 mm) and vegetable bases (963.8 mm). It indicates that the soil water leakage in the unsaturated soil under conventional cropped farmland increased significantly than the intensive cropped farmland although they have similar precipitation and similar irrigation case. It is because of the conventional cropped farmland lack of scientific management and control of irrigation under normal growing conditions. That results in wasting of irrigation water to a certain extent. However, the intensive cropped grape orchard and vegetable bases used a scientific and rational irrigation model to improve the utilization of irrigation water according to crop water demand characteristics. It reduced the amount of soil water leak effectively at different growth stages.

Table 3 also shows that the accumulated leachate of NO_3_-N within 0–100 cm soil profile in grape orchard is 277.1 kg/hm^2^, significantly higher than the vegetable bases and conventional cropped farmland areas, which are 91.3 kg/hm^2^ and 15.2 kg/hm^2^, respectively. It is mainly due to intensive application of organic fertilizer as basal fertilizer. Furthermore, there is some intermittent application of nitrogen fertilizers during the grape growing season. It resulted in long-term excess nitrogen accumulation in soil profile in the heavy rainfall. The strong leaching was caused by a large number of soil nitrate accumulation as rainstorm or irrigation happened. In contrast, intensive vegetable farmland deals with scientific methods to control water and fertilizer management of nitrogen fertilizer, effectively reducing the strength of the soil nitrate leaching; in conventional growing areas, due to the traditional model of low-intensity farming fertilization, soil nitrate accumulation is lower, which leads to a smaller amount of nitrate leaching.

According to the nitrogen migration characteristics and cumulative leaching loss amount, it can be concluded that the nitrogen leaching mainly takes place at the first stage of rainfall. In addition, differences in precipitation patterns have a significant influence on the amount of total nitrogen leaching out of the soil column, and thus rainfall intensity was as critical as the total amount of precipitation during the experiment. The results showed that the NO_3_-N leaching loss was affected by rainfall intensity and rainfall volume, and the NO_3_-N moved relatively easily with water in the soil profile.

### 3.3. NO_3_-N Leaching and Groundwater Quality

**Figure 9:**
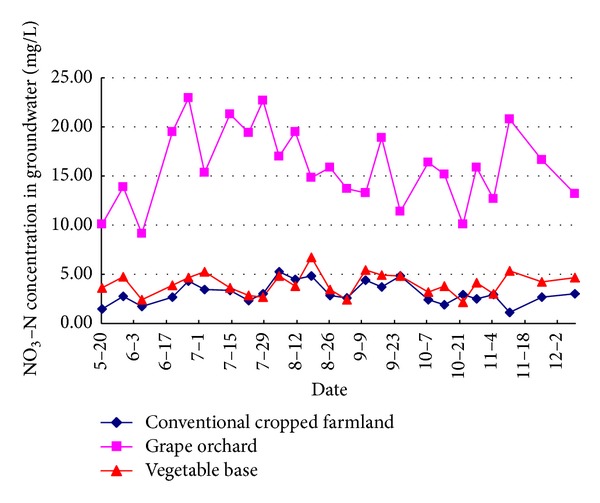
Change of NO_3_-N concentrations in shallow groundwater of three kinds of typical cropped soil.

The groundwater quality monitoring results indicate that the variation of NO_3_-N concentration fluctuated greatly in intensive grape orchard. Average concentration of NO_3_-N was up to 15.97 mg/L, of which the peak value of nitrate nitrogen reached 22.95 mg/L (Figure 9). The leakage of groundwater samples exceeded the NO_3_-N standard over a ratio of 100%, mainly due to high-frequency irrigation, fertilization in grape orchard. Moreover, long-term excessive fertilization also led to high nitrogen accumulation level in soil, and excessive irrigation increased the leaching degree of soil nitrogen. In the intensive vegetable farmland, the average groundwater concentration of NO_3_-N was 4.02 mg/L, while the peak concentration of NO_3_-N was high as 6.74 mg/L. It exceeded the standard over a ratio of 44%, indicating that during the cucumber-cabbage crop rotation period, there was little change in nitrate content of water leakage, and as a result there was minor agricultural shallow groundwater nitrogen pollution. Compared with intensive grape orchard and vegetable bases, the average groundwater concentration of NO_3_-N in conventional planted farmland was 3.54 mg/L. It exceeded the standard over a ratio of 20%, which was due to lower fertilization rates in conventional planted farmland. Figure 9 shows that the groundwater concentration of NO_3_-N in grape orchard was significantly higher than the level of intensive vegetable bases and conventional farmland, further indicating that excessive cropped farmland with intensive fertilization and irrigation on local farmland had resulted in shallow water environmental pollution hazards. Excessive irrigation and fertilization aggravated nitrogen leaching loss in intensive grape orchard, which induced NO_3_-N pollution in farmland groundwater environment. It proved that NO_3_-N in intensive planting areas had large and higher varied amplitude, and excessive irrigation fertilization had caused environment pollution of groundwater.

Table 4 demonstrates the statistical comparison of groundwater concentration of NO_3_-N in three cropped farmlands. Coefficient of variation in the conventional cultivation of farmland is high as 35.02%, while the coefficient of variation for NO_3_-N in intensive grape orchard and intensive vegetable bases is 24.48% and 27.91%. It is mainly due to disorder fertilization which is strong conventional cropped farmland. Average concentration of NO_3_-N in intensive vegetable base and conventional planted farmland is 4.02 mg/L and 3.54 mg/L, respectively, which are significantly lower than those in intensive grape orchard. According to the actual situation and management reality of the agricultural intensive planting areas, lots of controlling measures, such as optimizing irrigation and fertilizers, improving fertilization method to efficiently use fertilizer-nitrogen and reduce nitrogen leaching loss, and changing the traditional land use pattern, should be applied. The results in this paper also suggest increasing the efficient use of fertilizer-nitrogen, reducing nitrogen leaching loss, and putting forward feasible measurements for the management and control of nonpoint source pollution. It also provided the scientific basis for constituting the best management practices of the watershed. Research results were expected to provide scientific basis for optimizing field management practices, reducing farmland nitrogen leaching loss and controlling the agricultural nonpoint sources pollution.

### 3.4. Sensitivity Analysis

The model sensitivity analysis is helpful not only in reducing the complexity of the model and the workload of the data analysis and processing but also in improving greatly the accuracy of the model. The main factors which affect the LEACHM model for the water and nitrogen modeling are focused on the precipitation, soil texture, fertilizer types, and land use types. The accuracy of precipitation depends on the hydrological monitoring instrument. Saturated hydraulic conductivity (*K*) of soil water has obvious effects on the vertical migration of NO_3_-N. Water characteristic curve reflects the relationship between soil water suction and soil moisture. It also has a direct impact on NO_3_-N in the soil migration. Fertilization of different types and antimicrobial nitrification rate constants is different, and it will also affect the NO_3_-N vertical migration. Furthermore, dispersion can penetrate the soil solute some time earlier than some time later than the penetration, which reflects the soil large and small gaps in the distribution. Therefore, in this paper hydraulic conductivity *K*, water retention curve parameters *a* (*a* < 0), *b*, and nitrification kinetic constants *μ*, *m* are selected to do sensitivity analysis in LEACHM model.

**Figure 10:**
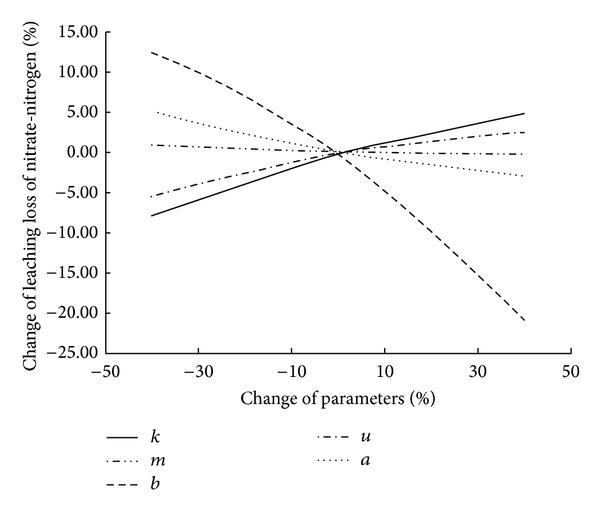
Sensitivity analysis for mainly parameters which affected the leaching loss of NO_3_-N in soil.

The sensitivity parameters based on the method of Lenhart [24] are classified as four types: (1) 0.00 ≤ |*I*| < 0.05, the low sensitivity parameters; (2) 0.05 ≤ |*I*| < 0.2, the middle sensitivity parameters; (3) 0.2 ≤ |*I*| < 1.0, high-sensitivity parameters; (4) |*I*| ≥ 1.0, super high sensitivity parameters. Five parameters sensitivity analysis results based on this method were shown in Figure 10. The calculated sensitivity index of the two parameters (*a*, *b*) of the water retention curve results were *Ia* = −0.096, *Ib* = −0.42, the sensitivity of hydraulic conductivity *K* index *Ik* = 0.16, nitrification kinetic constants sensitivity index *Iu* = 0.094, and dispersion sensitivity index: *Im* = −0.012. It can be concluded that water retention curve parameters *b* (*Ib* = −0.42) are high sensitivity parameters, a *a* (*Ia* = −0.096) is a sensitivity parameter, which affected the leaching loss of NO_3_-N in soil profile. Both of these two parameters showed a negative correlation between the amount of leaching loss and the change of water retention curve parameters. Hydraulic conductivity is the middle sensitivity parameters, and soil NO_3_-N leakage was a positive correlation when this parameter increased in the model, the NO_3_-N leakage with soil profile increased correspondingly. The nitrification kinetic constant is the sensitivity coefficient, and the NO_3_-N leakage with soil profile had a positive correlation between the amounts of leakage and this parameter. Dispersion is low sensitivity parameters, which was negatively correlated with NO_3_-N leakage within soil profile (Figure 10).

## 4. Conclusions

1. The LEACHM model proves to be capable of simulating NO_3_-N leaching loss in intensive agriculture cropped soil. Simulated results indicate that leaching amount of NO_3_-N within 0–100 cm soil profile is 277.1 kg/hm^2^ in intensive grape orchard during simulation period, but the leaching amount of NO_3_-N within 0–100 cm soil profile in intensive vegetable farmland and in conventional planting farmland is only 91.3 kg/hm^2^ and 15.2 kg/hm^2^, respectively. The leaching amount and the leaching loss rate of NO_3_-N in intensive cropped farmland are significantly higher than that of conventional planting areas. It shows that excessive fertilization increases the leaching risk of soil nitrate nitrogen and poses a potential threat to ecological environment in intensive grape orchard.
2. Monitoring results of groundwater quality showed that groundwater concentration of NO_3_-N in three typical planting areas is very large. The average concentration of NO_3_-N in grape orchard is the highest as 15.97 mg/L among three typical planted farmlands. It further suggests that NO_3_-N in intensive planting areas has large varied amplitude and higher over standard rate, and excessive irrigation and fertilization have caused environmental pollution of groundwater.
3. Sensitivity analysis demonstrates that water retention curve parameters (*a*) and nitrification kinetic constants *μ* are highly sensitive parameters, followed by the hydraulic conductivity (*K*), and the sensitivity is the smallest dispersion (*m*).
4. The work presented in this paper is also believed to be useful in formulating management strategies for intensive cropped catchment to reduce diffusive pollution from agricultural activities.

## Figures and Tables

**Table 1:** Selected physical properties and background values of soil samples.

Soil type	Depth (cm)	Bulk density (g·cm^−3^)	Soil porosity (%)	Organic matter content (%)	Soil texture (%)		
					Clay	Silt	Sand
Silt loam (Huangtu town)	0–20	1.16	55.56	2.48	22	46	2
	20–40	1.42	47.26	1.92	24	44	4
	40–60	1.49	44.79	0.58	24	44	4
	60–80	1.43	44.75	0.45	24	44	4
	80–100	1.40	44.74	0.33	18	50	4

Silt loam (Shengang town)	0–20	1.28	51.22	1.11	41	52	6
	20–40	1.30	51.88	2.11	43	49	6
	40–60	1.47	45.33	1.46	39	57	3
	60–80	1.49	44.79	0.36	26	67	5
	80–100	1.57	42.10	0.23	26	67	5

Silt loam (Xishiqiao town)	0–20	1.08	58.20	2.88	48	50	3
	20–40	1.37	48.91	2.43	50	48	3
	40–60	1.41	47.59	0.78	48	44	8
	60–80	1.41	47.49	1.50	46	43	10
	80–100	1.42	47.09	1.66	44	42	14

**Table 2:** Input parameter values used in the LEACHM model during calibration.

Parameter	Input values
Partition coefficient, NH_4_-N (m^3^/kg)	0.6 × 10^−3^
Partition coefficient, NO_3_-N (m^3^/kg)	0.55
Denitrification half saturation constant (mg/L)	10
Litter mineralization rate constant (per day)	0.01
Humus mineralization rate constant	7 × 10^−5^
Q10 factor	2.0
C : N ratio for biomass and humus	10.0
Maximum NO_3_^−^/NH_4_^+^ ratio in solution to control	8.0
Nitrification rate	100
Molecular diffusion coefficient (mm^2^/d)	140
Saturated hydraulic conductivity, (mm/d)	25
Water potential, kPa	1.3
Air-entry value, kPa	11.8
B parameters, kPa	

**Table 3:** Simulated soil water leaching and soil water storage variation under different planting systems.

Planted type	Precipitation + irrigation (mm)	Soil water leaching (mm)	Soil water storage (mm)
Grape orchard	1631.8	971.8	6.5
Vegetable base	1521.8	963.8	−46.4
Conventional farmland	1581.8	1177.6	−17.2
Planted type	Fertilizer usage (kg·hm^−2^)	Leaching loss for NO_3_-N (kg·hm^−2^)	Leaching rate for NO_3_-N (%)
Grape orchard	695.5	277.1	39.8
Vegetable base	381.4	91.3	23.9
Conventional farmland	178.6	15.2	8.5

Noted: + means that the soil water storage increases; − means that the soil water storage decreases.

**Table 4:** Statistical comparison of groundwater concentration of NO_3_-N in three different cropped farmlands.

Planted type	Average (mg/L)	Range (mg/L)	Coefficient of variation/(%)	Rate of exceed (%)
Grape orchard	15.97	9.10~22.95	24.48	100
Vegetable base	4.02	2.17~6.74	27.91	44
Conventional farmland	3.54	1.16~5.28	35.02	20
